# To Physicians and Druggists

**Published:** 1877-01-20

**Authors:** 


Business Manager.

TO PHYSICIANS AND DRUGGISTS.—The undersigned has connected with the journal business a purchasing agency in Atlanta, Ga., for the convenience of druggists and physicians, who can save time and traveling expenses by sending their orders direct to him. He has intimate acquaintance with the wholesale dealers in the city, and facilities which will enable him to select goods judiciously, and at the lowest rates.

To the friends and patrons of this journal no commission will be charged for this service.

Our readers will please direct the attention of their home druggists to the above agency.

Address DR. R. C. WORD,
Business Manager.

